# Effects of Mobile Health Including Wearable Activity Trackers to Increase Physical Activity Outcomes Among Healthy Children and Adolescents: Systematic Review

**DOI:** 10.2196/mhealth.8298

**Published:** 2019-04-30

**Authors:** Birgit Böhm, Svenja D Karwiese, Harald Böhm, Renate Oberhoffer

**Affiliations:** 1 Institute of Preventive Pediatrics Technical University of Munich Munich Germany; 2 Orthopaedic Hospital for Children Behandlungszentrum Aschau GmbH Aschau im Chiemgau Germany

**Keywords:** children, adolescent, mHealth, fitness tracker, physical activity, physical fitness

## Abstract

**Background:**

Children and adolescents do not meet the current recommendations on physical activity (PA), and as such, the health-related benefits of regular PA are not achieved. Nowadays, technology-based programs represent an appealing and promising option for children and adolescents to promote PA.

**Objective:**

The aim of this review was to systematically evaluate the effects of mobile health (mHealth) and wearable activity trackers on PA-related outcomes in this target group.

**Methods:**

Electronic databases such as the Cochrane Central Register of Controlled Trials, PubMed, Scopus, SPORTDiscus, and Web of Science were searched to retrieve English language articles published in peer-reviewed journals from January 2012 to June 2018. Those included were articles that contained descriptions of interventions designed to increase PA among children (aged 6 to 12 years) only, or adolescents (aged 13 to 18 years) only, or articles that include both populations, and also, articles that measured at least 1 PA-related cognitive, psychosocial, or behavioral outcome. The interventions had to be based on mHealth tools (mobile phones, smartphones, tablets, or mobile apps) or wearable activity trackers. Randomized controlled trials (RCTs) and non-RCTs, cohort studies, before-and-after studies, and cross-sectional studies were considered, but only controlled studies with a PA comparison between groups were assessed for methodological quality.

**Results:**

In total, 857 articles were identified. Finally, 7 studies (5 with tools of mHealth and 2 with wearable activity trackers) met the inclusion criteria. All studies with tools of mHealth used an RCT design, and 3 were of high methodological quality. Intervention delivery ranged from 4 weeks to 12 months, whereby mainly smartphone apps were used as a tool. Intervention delivery in studies with wearable activity trackers covered a period from 22 sessions during school recess and 8 weeks. Trackers were used as an intervention and evaluation tool. No evidence was found for the effect of mHealth tools, respectively wearable activity trackers, on PA-related outcomes.

**Conclusions:**

Given the small number of studies, poor compliance with accelerometers as a measuring instrument for PA, risk of bias, missing RCTs in relation to wearable activity trackers, and the heterogeneity of intervention programs, caution is warranted regarding the comparability of the studies and their effects. There is a clear need for future studies to develop PA interventions grounded on intervention mapping with a high methodological study design for specific target groups to achieve meaningful evidence.

## Introduction

### Background

Physical inactivity is an increasing public health problem among children and adolescents worldwide [1-3]. Only a minority meets the global recommendations of the World Health Organization (WHO) on physical activity (PA) for health [1,4-7]. Thus, young people aged 5 to 17 years should perform at least, in total, 60 min of moderate- to vigorous-intensity PA (MVPA) daily, including vigorous activities on at least 3 days per week [8]. Physical inactivity increases the risk of noncommunicable diseases [9], already for primary school children [10], and represents the fourth-largest risk factor for mortality in the world [11].

Intervention strategies for health promotion, especially in children, must start early to grow healthy into adulthood because health-related attitudes and behavior patterns develop in early childhood, which are often maintained up to adolescence and adult age (12 to 14 years) [12]. Regular PA makes a significant contribution to the positive development of health in childhood and youth [4]. Numerous health benefits of regular PA are plentiful in this age [13], which persist into adulthood, such as positive effects on fitness, body fat, and blood pressure [14]. The dose-response relations observed in observational studies indicate that the more PA, the greater the health benefit. To achieve substantive health benefits, PA should be of at least a moderate intensity. Vigorous intensity activities may provide even greater benefit. Aerobic-based activities had the greatest health benefit, other than for bone health, in which case high-impact weight-bearing activities were required [15]. However, nowadays, an inactive everyday life is already ubiquitous in a young age [16,17].

The development of effective interventions to encourage active lifestyles among children and adolescents is one opportunity to address the lack of PA in this population group [18]. Use of technology-based interventions makes it more interesting [19]. Some preliminary data suggest that wearable activity trackers may have the potential to increase activity levels through self-monitoring and goal setting in the short term [14]. Mobile health (mHealth) and wearable activity trackers represent 2 of these innovations.

To date, a standardized definition of mHealth is not established. This is demonstrated in the fact that the terms *mHealth*, *electronic health (ehealth)* and *telehealth* are frequently used interchangeably [20]. The definition of mHealth as medical and public health practice supported by mobile devices, such as mobile phones, and other wireless devices is taken by WHO [21].

The use of smartphones among young people has increased in recent years. One-fifth of Germans aged 6 to 7 years use a smartphone. From the age of 12 years and above, the usage is over 80%. Tablets are most commonly used by those aged 12 to 13 years (43%) [22]. This trend can also be seen in the United States [23,24] and also in developing countries [25] where smartphones are used more than any other modern technology. Therefore, mobile devices and apps may be an effective strategy for promoting PA in this target group.

Furthermore, there is an increasing interest in commercial wearable devices that track health- and fitness-related activities and promote PA [26]. On the basis of their growing availability, popularity, and widespread adoption, they also offer a creative solution for children and adolescents to get moving in a playful way [23]. Currently, there are no data available on how many children and adolescents use wearable activity trackers. However, wearables are increasingly gaining importance as smart gadgets, and manufacturers are always looking for new apps to increase their sales [24]. Fitness trackers, such as *Garmin vívofit jr.* and *Jawbone UP*, are used to promote PA among this target group [27,28]. The former was developed for children and also involves the parents by setting tasks and defining rewards [27].

To date, several reviews have mainly or partially focused on PA outcomes of mHealth tools, in which mostly studies with adults were examined [29-32]. A large volume of PA research with tools of mHealth has primarily focused on weight control (eg, to prevent obesity) [33,34], on the treatment of diseases (eg, chronic diseases such as diabetes mellitus) [35,36], or on improving medication adherence [37,38].

The number of reviews on the issue of mHealth on PA is still low, compared with reviews using wearable activity trackers to increase PA. To date, wearable activity trackers have been primarily studied to examine their ability, validity, and reliability to estimate PA [39,40]. There are also some feasibility studies of these devices for children [41,42]. Frequently, they are used in an intervention as an evaluation tool to measure PA levels objectively [39,43]. However, little is known about the effectiveness of these devices as a tool for promoting PA outcomes, whether as a single strategy or in combination with others. Until now, healthy children and adolescents seem to play a tangential role in this area of research. The review by Lewis et al [44] reported some initial evidence that wearable activity trackers can increase PA, but only studies with adults have been taken into account. However, children and adolescents have a high affinity to new technologies and use them in their daily lives.

### Objectives

To date, no review evaluated tools of mHealth as well as wearable activity trackers that promote PA and increased PA behavior in healthy children and adolescents. Therefore, there is a research need to evaluate systematically whether tools of mHealth or wearable activity trackers are appropriate and effective in promoting and changing PA in this target group. The results are important for informing and supporting future PA interventions in young people. Moreover, it has the potential to contribute to the development of public health guidelines relating to the role of these tools in PA and health promotion.

Therefore, the aim of this systematic review was to examine the effectiveness of interventions that use tools of mHealth, respectively wearable activity trackers, to promote and change PA among children and/or adolescents. As the health status may confound the effectiveness of interventions this review considered only healthy children and adolescents.

## Methods

### Treatment Objectives

The definitions and inclusion criteria used are described below.

#### Mobile Health Tools

Mobile phones, smartphones, tablets, and apps on these devices were considered as tools in the field of mHealth, which aimed to promote PA to increase PA levels among healthy children and adolescents. With regard to the widespread usage, the field of mHealth is already strongly focused on these devices, especially on smartphones, to create promising interventions for the youth [45-50].

#### Wearable Activity Trackers

Wearable activity trackers were defined as an electronic device with the following features: designed to be worn on the user’s body; uses accelerometers, altimeters, or other sensors to track the wearer’s movements or biometric data or both; and can provide feedback via the monitor display or through a partnering app to elicit continual self-monitoring of activity behavior [44,51]. This definition eliminates pedometers and accelerometers that do not supply automated feedback to the wearer [44]. Systems with feedback were included in the definition, as self-monitoring resulted in significantly more activity compared with a no-feedback condition [52-54].

#### Data Source and Search Strategy

A systematic literature search was conducted to find out relevant articles in 5 electronic databases: the Cochrane Central Register of Controlled Trials (CENTRAL), PubMed, Scopus, SPORTDiscus, and Web of Science. The search was conducted according to the Preferred Reporting Items for Systematic Reviews and Meta-Analyses (PRISMA) guidelines. For most entries, text words and synonyms were used, marked with (tw), plus Medical Subject Headings (MeSH), marked with (MeSH), for major records with relevant keywords without any limitations. The MeSH search was only conducted in CENTRAL and PubMed databases (Multimedia Appendix 1). Accordingly, text words were oriented on entry terms of MeSH headings to cover a comparable search spectrum in the other data banks this was especially necessary in the German data bank, SPORTDiscus, as these conceptually, for example, put another focus (Multimedia Appendix 2).

Search strategies for the various databases contained search strings in 4 main areas: population, treatment method, treatment objective, and outcome variable. Terms for mHealth and wearable activity trackers were adapted from previous reviews [14,38] and entry terms from the MeSH heading *Fitness Trackers*, which were introduced in PubMed in 2017.

The search was carried out based on article title, abstract, and keywords in all 5 databases. In the case of PubMed, the terms were entered into the search box using PubMed’s search field tags (tw) for the text words and their synonyms and (mh) to search the MeSH headings. In all databases, each individual term was scanned first. Then, the Boolean operator *OR* was used within the individual areas and, subsequently, the operator *AND* to combine the 4 search fields. Finally, the available limits in the various databases were selected.

The reference list of thematically related review articles was also searched for potentially useful sources. Checking the bibliographies of identified studies is a generally used approach to identify additional relevant studies for potential inclusion in systematic reviews [55].

### Selection Criteria of Studies

#### Inclusion Criteria

1. Published in peer-reviewed journals in English. Studies in press were included if they had a unique digital object identifier.
2. Published from the beginning of January 2012 to the end of June 2018.
3. Focused on children and/or adolescents.
4. Included healthy participants (including underweight, overweight, and obese without any reported dysfunction).
5. Specifically examined the use of at least 1 mHealth tool or of a wearable device within an intervention to promote PA (even if it was only 1 component of the whole intervention).
6. Measured at least 1 PA-related variable as the outcome (in this, connection was not defined as a restriction regarding the types of PA-related outcomes, which could be cognitive [ie, PA knowledge and PA self-efficacy], psychosocial [ie, PA intention, social support to PA, and stage of change], or behavioral [ie, energy expenditure, step counts, or observed or self-reported PA level], or physical fitness).

Overall, randomized controlled trials (RCTs) and non-RCTs, cohort studies, before-and-after studies, and cross-sectional studies were considered. If the study design was not clearly stated but contained in their description characteristics of one of the included study designs, it was included. In addition, studies based on an experimental design were checked according to this criterion and upon fulfillment were included. If there were multiple publications from mHealth or wearable activity tracker interventions, only the study with the PA outcome(s) or the most recent publication with PA outcome(s) was included.

We oriented the relatively strict search years at the time when consumer wearable activity trackers with proofed validity and reliability entered the market following the review of Everson et al [39]. In addition, the results of Ridgers et al [14] were taken into account, as well as the review on mobile phone interventions published in 2017 [56].

#### Exclusion Criteria

1. Conference proceedings, book chapters, dissertations, pilot studies, and systematic reviews.
2. Studies where the main mHealth component was not mobile, eg, Web- or email-based), that did not evaluate at least 1 mobile aspect of assessment or intervention delivery, or where there were no indications of mobile platform compatibility (eg, the app used on a desktop computer does not run equally on a tablet or smartphone).
3. Studies that used wearable activity trackers only to evaluate an intervention.
4. Articles examining the validity or feasibility of mHealth tools or wearable activity trackers were excluded if they did not evaluate these as technologies to measure participants’ PA-related outcome(s).
5. Studies where participants had additional reported dysfunctions.
6. Studies focusing on weight control or loss without any PA measurement.

### Study Selection and Data Extraction

Following a standard protocol, 2 authors (SS and BB) independently screened studies for eligibility based on the title, abstract, and full text. Uncertainty was discussed involving a third author (RO), and any disagreement was resolved by consensus.

**Figure 1:**
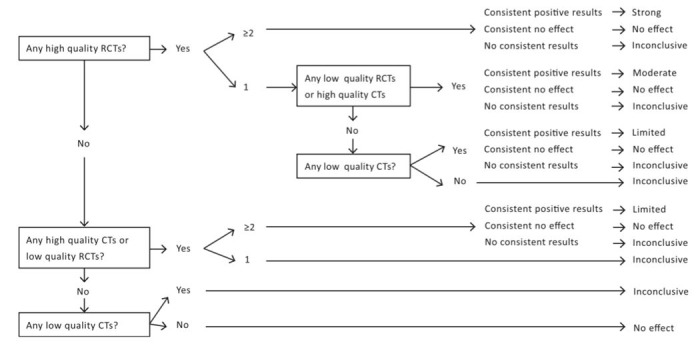
Flowchart of the decision-making process for levels of evidence, based on study design and methodological quality. CT: controlled trial; RCT: randomized controlled trial.

All search results were exported into EndNote X7.7.1 (Thomson Reuters). Information about each paper was extracted by BB and SS independently for quality assurance. Screening of all entries took place in 4 steps: First, duplicate references were removed. Then, all titles were screened, and additional entries, which did not match the MeSH terms and text words that lead to a different content, were removed. Entries were left in the database if the context was not fully clear from the title. After that, abstracts of the remaining articles were screened. If there was any doubt in the information in the abstract, the full article was retrieved to ensure that no relevant entries were lost. In the end, full text articles were retrieved for further assessment, if the eligibility criteria had been fulfilled or suggested that the article was a potential study for this review. All the remaining entries were reviewed for final inclusion. Figure 1 contains the excluded criteria for decision making of the selection of potential useful articles used in the examination of the abstracts and full texts.

The data from the selected intervention studies were extracted with regard to the following information: (first) author; year of publication; study design; country in which the intervention was carried out; place of recruitment; number of study participants and their characteristics (age, gender, and body mass index [BMI]); types of tools used (in the field of mHealth or wearable activity trackers); intervention description and duration; time of measuring and measuring instruments of PA-related outcome(s); and key findings on PA or physical fitness. In the case of a controlled trial (CT) with comparison between the intervention group (IG) and control group (CG), further data were also extracted: task or program of CG and differences in PA levels between the IG and CG.

**Figure 2:**
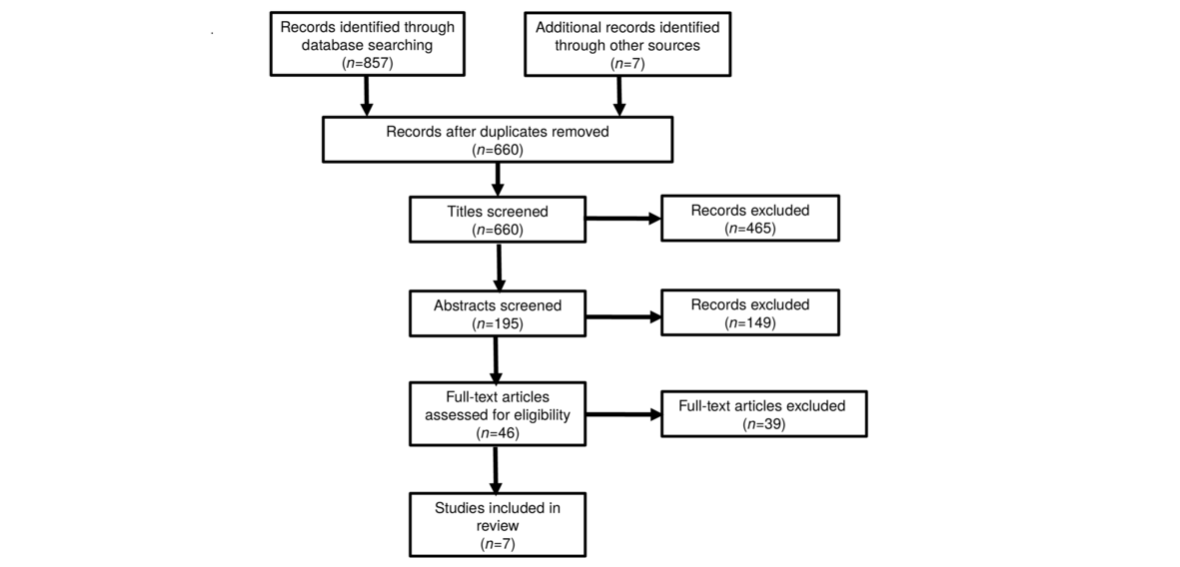
Process of identification and selection of included studies. Databases used were Cochrane Central Register of Controlled Trials (n=136), PubMed (n=88), Scopus (n=216), SPORTDiscus (n=199), and Web of Science (n=211).

Furthermore, studies were distinguished by their intervention field: (1) tools of mHealth or (2) wearable activity trackers. A PRISMA flow diagram presents the summary of the study selection process (Figure 2). Databases used were Cochrane Central Register of Controlled Trials (n=136), PubMed (n=88), Scopus (n=216), SPORTDiscus (n=199), and Web of Science (n=211). At abstract screening, records could be excluded for at least 1 of these reasons (the first exclusion criterion was always counted, even if several apply): (1) no study with use of at least one mHealth tool or a wearable activity tracker to promote PA (n=84); (2) no target age groups (n=46); (3) no healthy participants (n=1); (4) no original study (n=15); (5) no measurement of at least one PA variable (n=2); or (6) not published in a peer-reviewed journal (n=1). At the full-text article screening, studies were excluded for the following reasons: (1) no study with use of at least one mHealth tool or a wearable activity tracker to promote PA (n=17); (2) without PA measurement (n=5); (3) duplicate publication (n=1); or (4) not selected study design (n=10). Additionally, 1 full text could not be obtained, and for 5 studies, it was only revealed in the full text that the participants did not meet the age criteria groups (n=3), were not healthy (n=1), or it was a previous publication (n=1).

### Publication Bias

A sufficiently large number of studies will be included in this review (including some with high subscriber numbers), funnel-plot analysis will be implemented. This is used as a test for publication bias and similar systematic errors [57].

### Criteria of Methodological Quality

Only controlled studies with PA comparison among groups were examined with regard to their methodological quality. It is essential to take into account a possible biasing influence on estimates of intervention effectiveness [58]. By means of the tool by the *Cochrane Handbook for Systematic Reviews of Interventions*, systematic reviewers are provided help to select the adequate criteria for evaluating the possible bias in a specific field of study. These standards were primarily developed for medical and health science studies [59]. On the basis of these recommendations [59] as well as previous systematic reviews that can be applied to this research field [60-62], a total of 9 criteria must be satisfied to maintain high methodological quality (Multimedia Appendix 3). One point was given to a study if a criterion was met, whereas no points were given when a criterion was not fulfilled or when it was not (sufficiently) described. A methodological quality score (ranging from 0 to 9) was calculated by accumulating all positive items. Studies scoring 0 to 2 points were of low methodological quality, studies with 3 to 5 points were of moderate quality, and studies scoring 6 or above were of high methodological quality. If the study was a non-RCT, the score had to be at least 5 for high methodological quality (owing to the fact that 1 item was regarded to the randomization procedure).

With the aid of the selected quality characteristics, the risk of the 4 most important forms of bias could be examined, which could influence the (internal) validity of a study: selection bias, performance bias, measurement bias, and attrition bias [57].

### Strength of Evidence

The strength of evidence was evaluated based on a previously used evidence synthesis method [60,63,64]. Therefore, the effects of interventions with the use of tools of mHealth or wearable activity trackers on PA were rated using an evidence rating system adopted from a study by Liang et al (Figure 2) [62]. As a result, the following 5 levels were defined based on the study design and methodological quality: (1) strong; (2) moderate; (3) limited; (4) inconclusive; and (5) no effect. The studies were stratified based on their intervention tool: in the field of mHealth or wearable activity trackers. Following a review by van Sluijs et al, the overall results were considered as consistent if at least two-thirds of the relevant studies had significant results in the same direction [60].

## Results

### Included Studies

In total, 864 records were found through a systematic search of 5 databases and other sources that were thematically related reviews and retrieved studies. Finally, 7 trials were identified matching the inclusion criteria (Figure 1), of which 5 used a tool of mHealth [65-69] and the other 2 studies made use of wearable activity trackers to promote PA among children and/or adolescents [70,71]. All 7 studies are described in detail, distinguished by their intervention field: tools of mHealth (Multimedia Appendix 4) and wearable activity trackers (Multimedia Appendix 5). For the description of the intervention characteristics, the study protocols of 2 included studies were additionally consulted [72,73].

The designs of the intervention programs were guided from theoretical frameworks and behavior change techniques (BCTs). Some were referred only to one theoretical model, but most of the studies integrated more than one theoretical model. As basic theoretical model, the *Social Cognitive Theory* by Bandura was most frequently used [65,68-70]. In total, 3 studies integrated additional BCTs [65,66,68] such as feedback on behavior, self-monitoring behavior, goal setting, and strategies to overcome barriers. Except for 2 studies [66,71], no information was given.

The variety of and the inconsistency in the methods used for data assessment across the studies make it difficult to compare the effects on PA. Accelerometers were used predominantly for objective PA measurement; however, the inclusion criteria varied for the data to be included in the evaluation. Participants’ data were generally included in the analyses if valid data for at least 3 days existed. Only the study by Dewar et al requested an additional weekend day [65]. However, wearing time of the monitors varied. In total, 2 studies determined a wearing time of at least 600 min per day [65,66], whereas others demanded ≥480 min per day [67,68]. To evaluate the PA self-efficacy, for example, Direito et al used the *Physical Activity Self-Efficacy Scale* questionnaire [66]; however, Dewar et al designed their own questionnaire that inquired self-efficacy as one item [65].

### Publication Bias

This review included only a small number of studies (*n*=7), so the presentation of the funnel plot was omitted. Therefore, the existence of publication bias is to be assessed as unclear.

### Methodological Quality

Multimedia Appendix 6 reports the methodological quality for the 5 selected intervention studies with group comparison [65-69]: 4 were designed as RCTs [65,66,68,69] and 1 was a randomized controlled cross-over trial [67]. In the case of the study by Garde et al [67], it was necessary to consult the study protocol to clarify whether the item (9) was met [74]. In total, 60% of the studies (*n*=3 studies) were of high methodological quality [65,66,68] and 2 studies of moderate quality (40%) [67,69]. Lubans et al reached the highest score [68]. Their study met all the criteria except for the blinding criterion, which, however, was also not fulfilled by any other study. However, the application of blinding strategies in mHealth is often impossible, impractical, or infeasible, thus making blinding more difficult [75].

To obtain a more differentiated insight into the intervention studies, each methodological criterion was examined on its own (Multimedia Appendix 7). All studies adequately carried out pretest analyses, met the criterion of the timing of measurement, and used valid measurement instruments. In most cases, accelerometers were applied to measure PA levels objectively [65-68], whereby the *ActiGraph* accelerometer was most commonly used [65,66,68]. In 3 out of 7 studies, the assessors of the pretest were not blinded [65-67], and the other 2 studies had no sufficient information about their blinding process [68,69]. The randomization criterion was met by 5 out of 7 studies. Although Zach et al divided their participants randomly, they did not explicitly describe the method used [69], and Garde et al included only 42 participants in their randomization process [67]. A clear randomization process at the level of experimental planning is necessary to ensure that potential confounders are evenly distributed among the comparison groups. Through randomization, a relation between potential confounders and the exposure can be excluded [76]. The criterion of dropout rate was met by 2 out of 7 studies, and the criterion of systematic dropout was met by 3 out of 7 studies. Only the study by Lubans et al [68] included a follow-up measurement realized at a minimum of 3 months after completion of the intervention. Finally, the criterion of the sample size was met by 4 out of 7 studies. Thereby, 2 studies examined samples larger than 250 participants [65,68] and the other 2 had smaller sample sizes but carried out a power calculation [66,69].

### Strength of Evidence

In total, 5 studies used a tool of mHealth to promote PA, including 3 high-quality RCTs [65,66,68] and 2 RCTs with moderate methodological quality [67,69] with one of those designed as a cross-over study [67]. The 3 high-quality RCTs with an objectively measured or a self-reported PA level, that is, MVPA, consistently reported no statistically significant effects on their PA outcome. This means that there was evidence of no effect on PA-related outcomes among interventions with tools of mHealth.

Both the intervention studies with wearable activity trackers were designed as before-and-after trials [70,71]. According to the flowchart (Figure 2), only a low-quality CT was available. Therefore, no effect of wearable activity trackers on PA was identified.

To summarize, no evidence of an effect among all interventions with tools of mHealth or wearable activity trackers, or both to promote PA among healthy children and/or adolescents was identified.

### Intervention Studies: Tools of Mobile Health

#### Text Message

Only the study by Dewar et al used short message service (SMS) text messaging as one element in their intervention program [65]. In total, 8 different components were integrated in their study [72]. The overall aim was promoting PA and healthy eating and preventing obesity in female adolescents. The participants (*n*=357 girls; mean age 13.2 [SD 0.5]) came from economically disadvantaged secondary schools (*n*=12) and were disengaged in physical education (PE) and/or not currently participating in organized team sports or individual sports. With 12 months, this study had the longest intervention period. The primary outcome (BMI) was reported in a previous publication [77]. In this study, the secondary outcomes (ie, objectively measured PA with *ActiGraph* accelerometers) were examined. According to the study protocol, the text messages were sent to the participants each morning during the 7-day monitoring period to remind wear and improve compliance. However, the study did not report this step.

After 12 months, PA data from 246 girls could be evaluated. There were no significant group-by-time effects for moderate PA (MPA) and vigorous PA (VPA), as well as MVPA. Changes for most of the social-cognitive variables were found in the IG. However, there were no statistically significant effects. Follow-up data were not published. Only baseline results and measures after 12 months were analyzed.

#### Smartphone App

Direito et al investigated the effects of 2 commercial smartphone apps (*Zombies, Run!* as an immersive app and *Get Running* as a nonimmersive app) [66]. The participants (*n*=51; mean age 15.7 [SD 1.2]) owned an iPod touch or smartphone running at least iOS 6.0 or Android 2.2, respectively. In relation to PA, they were able to perform PAs but were not achieving the PA recommendations of their age group. The apps served to improve PA as well as the ability to run 5 kilometers. Participants received gift cards to a local shopping center for each visit to complete study measures independent of their usage of the app. PA levels were evaluated by self-reporting and measuring using the *ActiGraph* accelerometer. In addition to cardiorespiratory fitness and PA outcomes, the features of the app design were also evaluated with regard to their acceptability and usability.

After 8 weeks, no significant increases for self-reported PA and PA self-efficacy were recorded. In addition, there were no statistically significant effects on physical fitness. The average daily time spent in MVPA had a decrease toward the baseline in the *Zombies, Run!* group and the CG (posttest 33.04 signified 55% to daily recommendations [8] and 30.54 signified 51% to daily recommendations) [8]. The *Get Running* group reported an increase from 21.29 to 23.34 (signified 38.9% to daily recommendations) [8]. This group was overall the weakest in terms of PA-level measurements.

In Canada, Garde et al examined the efficacy of the mobile exergame *MobileKids Monster Manor* (MKMM) in a school-based environment (*n*=42; mean age 11.3 [SD 1.2]) [67]. The most innovative and special feature of MKMM was that the player had to earn in-game playtime by performing PA.

After 4 weeks, they could confirm their hypothesis that children with a higher BMI *z*-score had greater benefit while playing the game. The increase in PA was significantly greater relative to their counterparts with a lower BMI *z*-score (*P*<.05), which was also observed in Garde et al previous community-based study [74]. Furthermore, there was a significant PA difference between the game intervention and control weeks, showing more steps (*P*<.001) and active minutes (*P*<.001) per day during the intervention. PA was recorded using the *Tractivity* activity tracker, which considers an active minute only if it contains at least 20 steps within a window of 7 active min. In addition to PA, the experiences playing the game were evaluated using a survey. In total, 90% of the children thought that MKMM was very effective at promoting PA.

Lubans et al were the only group that evaluated an 18-month follow-up [68]. This study was designed to be culturally appropriate and incorporated mHealth technology with the goal of preventing obesity among adolescents [73]. The participants were disadvantaged boys failing to meet the international PA guidelines, why they were *at-risk* of obesity, whereby the weight status was not an inclusion criterion (*n*=361; mean age 12.7 [SD 0.5]). The study included 7 components (ie, a smartphone app) and involved teachers, parents, and students. After the study endpoint, participants still had access to the smartphone app.

After 18 months from baseline, significant intervention effects for PA were not reported (*P*>.05). The differences at baseline between completers and dropouts for the outcomes at 18 months were not meaningful.

Zach et al included only female high school students in their program, which was carried out during PE lessons, and measured psychological and physiological effects (*n*=154; ages 16 to 18 years) [69]. The reason for the focus on female subjects was that PE in grades 7 to 12 is single sex and not coeducational, and the lack of motivation to participate in PA in leisure time is also more prominent in Israel among female adolescents. In 1 IG, they used the smartphone app, *WhatsApp*, so that the participants could write a short personal report to the class *WhatsApp* group. This report was also shared with the teacher. Overall, they chose the internet and the app as 2 different kinds of technical methods to evaluate which of the two would serve as a better means for also increasing self-efficacy for independent PA.

At the end of the 12-week intervention, the results of the participants’ perception of self-efficacy for independent training were inconsistent. Predominantly, no prepost differences or interactions were observed. Zach et al also hypothesized that the best IG would have the greatest significant benefit in physical fitness. But significant differences between groups showed up in all physical fitness measures (*P*<.05).

### Intervention Studies: Wearable Activity Trackers

Bronikowski et al used the *Garmin vívofit* activity tracker (model not reported) as an evaluation and intervention tool to examine the effectiveness of different target strategies on PA behaviors among children and adolescents (*n*=193; aged 11 to 17 years) [70]. The *Goal set* group had a daily goal of 10,000 steps for adolescents and 12,000 steps for young adolescents and children. The *Do your best* group did as many steps they could and wanted to do daily. The activity tracker was worn during the whole intervention. All participants could see the number of steps and had an internet account created on the *Garmin Connect* program to follow their progress and weekly trends. The level of PA was determined by means of a PA screening measure, and an MVPA index was thus calculated. The average number of steps was only identified in the posttest. In addition, classmate and teacher support in MVPA during PE lessons was evaluated.

At the end of the 8-week intervention, all adolescents of the 2 groups achieved 10,000 steps, whereas all young adolescent girls could not reach their recommendation of 12,000 steps. In comparison with this, in the group of children, only the girls from the *Do your best* group met the criterion. Generally, in most cases, the daily average was higher in the *Do your best* group. However, the MVPA index decreased in this group. The external support of classmates and teachers was not taken into account in this review as sufficient information about the exact actions in which this support should promote PA was not given.

In addition, Hayes and Van Camp applied an activity tracker to promote PA [71]. They used *Fitbit* (model also not reported) as a tool to increase the PA levels of girls in the third grade in an elementary school (*n*=6; aged 8 years) during unstructured school recess as well as their evaluation tool. During the baseline process, the participants did not receive feedback regarding their number of steps. The criterion for moving out of baseline was stability or the absence of an upward trend. After baseline data were collected from 7 recess periods, girls were provided with step goals for 7 further recess periods. In addition, they were encouraged to self-monitor their steps against goals. The incremental increase was based on the baseline data. Subsequently, data were collected for a further 7 periods, in which there were no step goals provided. For the final intervention session, 3 goals were given (20%, 30%, and 40% increase). Moreover, a tangible reward (eg, small toys) was provided based on the goal(s) achieved. Results revealed an increase in steps by 47% from baseline, which contributed 18% to the daily step recommendations [78]. The percentages of time spent in MVPA increased from 4% (range 2% to 6%) to 25% (range 10% to 41%), which equate to 5 min of MVPA during recess or a contribution of 8% to the WHO’s daily recommendations of at least 60 min MVPA [8]. Without the use of the *Fitbit* activity tracker to self-monitor recess activity, the number of steps and MVPA decreased to initial baseline levels.

## Discussion

### Principal Findings

In total, 7 intervention studies were identified that reported the use of mHealth tools or wearable activity trackers in healthy children and adolescents with PA-related outcomes. Most of them (5/7, 71%) included mHealth technologies such as mobile apps, games, and SMS text messaging in efforts to promote PA among children and/or adolescents [65-69]. None of the studies had the same mean age of the trial population, which ranged from 11 to 18 years. The study results of the 3 high methodological RCTs consistently reported no statistically significant effects on their PA-related outcomes. However, there was evidence of no effect in relation to the applied scheme. It should be noted that in relation to smartphone apps, 81% were interested in trying various PA-promoting apps in the future [66] and 92% of the children in the study by Garde et al enjoyed the requirement of being active [67]. Games in the form of mobile apps seem to be an attractive tool to promote PA among the youth. However, game design should be appropriate for specific age groups.

Only 2 studies using wearable activity trackers met the criteria to be included. In these studies, the activity trackers were used as the intervention as well as the evaluation tool to measure MVPA [70,71]. Neither of them used an RCT but used a before-and-after study design. On the basis of the applied scheme by Liang et al [62], there was no effect of these devices on PA.

### Intervention Approaches

Most of the included studies were set in a school environment. The importance of schools as a setting in PA promotion has already been highlighted [79]. Children and adolescents spend much of their time there, so this environment offers itself to implement such interventions aimed at promoting PA [80].

The studies had different restrictions on the PA of their included participants. Examining the PA status (time spent in VPA, MPA, and MVPA) to select suitable participants is useful. For example, Bronikowski et al reported that their subjects had a reasonably high level of MVPA before their intervention, which is why their effects could have been weakened [70]. Therefore, the approach of some studies focusing on healthy children and adolescents who did not meet their PA recommendations by WHO appears appropriate [66,68]. Moreover, PA intervention programs focusing on children and/or adolescents with a greater BMI *z*-score are necessary. Garde et al could affirm their hypothesis that they have a greater benefit on PA [67]. In total, 2 studies recruited their participants from socioeconomically disadvantaged schools and focused on a specific gender [65,68]. In addition, Hayes and Van Camp and Zach et al included only female participants [69,71]. It is known that especially girls with a low socioeconomic status showed a lack of PA [81]. Therefore, sex-specific interventions should be considered in future research.

### Intervention Studies with Tools of Mobile Health and Their Effects

First, all studies with tools of mHealth were designed as RCTs. Second, except for 1 study, where no sufficient information was given [66], the intervention programs were grounded on theoretical frameworks. Third, most of the studies also integrated BCTs [65,66,68]. BCTs are defined as an observable, replicable, and irreducible component of an intervention program designed to change or redirect causal processes that regulate behavior (eg, feedback and self-monitoring) [82]. If an intervention’s description of a study named their BCTs, the effectiveness of the intervention can also be associated with these strategies [82]. For example, the apps used by Direito et al included self-regulatory BCTs (ie, prompt specific goal setting, prompt self-monitoring, and provision of feedback on performance) [83]. However, no significant effects were reported.

The limited significant effects observed in intervention studies with tools of mHealth included in this review may be the result of the intervention design and the PA evaluation method that has been elaborated below:

First, the intervention duration of most studies lasted from a minimum of 4 weeks to a maximum of 12 weeks. Nguyen et al reported in their review published in 2016 that behavioral interventions with a duration of ≥6 months had greater success in changing PA levels [84]. Thus, it is possible that a short period of time could be insufficient to change behavior. However, Dewar et al delivered their intervention over 12 months, but did not find significant effects [65]. However, their main aim was to reduce the BMI; thus, it is possible that the components of their program were not sufficient for changing PA positively. There is a need for PA studies with comparable long intervention durations to find out whether significant effects are found.

Second, only 2 studies involved a large number of participants (≥250) [65,68], so most of the results may not be representative for the total population.

Third, researchers did not supervise the intervention programs, with the resulting limitation not guaranteeing the extent to which the intervention measures were implemented [85]. For example, Dewar et al had information that 91% of the IG accessed text messages, but it was unknown if these were read by the participants [65]. Lubans et al did not have objective usage data to determine participants’ continual engagement with the smartphone app [68]. In addition, Direito et al did not closely monitor use of the apps during their intervention [66]. Indeed, they wanted to show the usefulness of the apps in real life [66]; however, important data are missing for evaluation. These examples show how essential it is to verify the intervention components to draw conclusions about their effectiveness.

Fourth, a general problem was the lack of compliance. All studies with the use of an accelerometer reported failing compliance [65-68]. On the basis of insufficient wear time, the inclusion criteria to evaluate PA were not met by the majority of subjects. Only 24.6% met the criteria by Dewar et al at posttest [65]; Lubans et al included only 32% of their participants [68]; and Garde et al received valid data from 28 out of a total of 42 participants (66.7%) [67]. In comparison with this, Direito et al had a low failure of data (loss of 4%) at posttest [66]. However, the reduced number of cases in most of the studies resulted in underpowered analyses, so their findings are not meaningful enough.

Fifth, 3 studies used questionnaires for PA-related outcome measurement [65,66,69]. However, self-reported measures are vulnerable for recall as well as report bias, which is also susceptible for social desirability bias.

Finally, 80% of the included studies used accelerometers to measure PA levels objectively [65-68]. These devices have adequate reliability for PA surveillance. However, there still exist several issues associated with their validity [86]. Accelerometers lack the sensitivity to recognize and record nonambulatory movements, so not all forms of movement are detected [65]. These restrictions could have led to the reported lack of intervention effects.

In conclusion, future research projects in this field are encouraged to develop intervention programs with a longer period of time (≥6 months), including a sufficiently large number of participants (≥250) to receive meaningful results about their efficacy. Moreover, bias should be avoided. For this reason, self-reported measurements should be bypassed. Furthermore, measuring instruments should be checked in advance for their specificity. Owing to the preferable use of accelerometers to determine PA, despite their poor compliance among the youth, further investigations to improve this are indicated.

Future intervention programs should use the intervention mapping (IM) by Kok et al to develop theory- and evidence-based health promotion interventions [87] to be able to assess the impact these factors (eg, election of BCTs, intervention components, and implementation of the program) may have had on the overall findings.

### Intervention Studies With Wearable Activity Trackers and Their Effects

Although 1 study was able to observe a clear percentage increase in the number of steps during their intervention phase (increase of 47%), there was a lack of statistical evaluation. Another limitation was that some data for MVPA calculation were lost because of syncing failures, including the last intervention session. In addition, the increase in MVPA during brief 20-min sessions was not consistent and may not currently suffice for clinical significance as well as for a transfer into practical recommendations [71]. Overall, it can be concluded that there were limited intervention effects in the studies with wearable activity trackers as their intervention tool. This result may be attributable to several factors as follows:

First, the intervention duration amounted to a maximum of 8 weeks [70] which might be too short to change behavior [84]. There exists a clear need for studies using longer intervention periods to obtain more meaningful results regarding the effectiveness of these devices in the youth. Second, Hayes and Van Camp had recruited a small number of children; therefore, the study has been underpowered to detect a significant change in PA [71]. Third, in this study, it was not reported if the intervention was grounded on behavioral theories [71] that are essential for intervention effectiveness [88]. Fourth, the selection of the participants should be characterized with regard to their PA level in advance. Bronikowski et al assumed that the missing significant intervention effect might have resulted from MVPA of the participants already present before the beginning of the intervention [70]. One possibility would be to include children and/or adolescents who are healthy but do not meet the recommendations of PA. Fifth, the studies used the data of their wearable activity tracker to measure MVPA. However, it has been noted that, to date, these devices are not validated for assessing PA-related outcomes in the youth [39]. As long as this restriction still exists, the intervention effects should be recorded using validated objective monitors such as accelerometers. It is further important to pay attention to compliance. Finally, the school setting in which the interventions were implemented could influence the study results. Schools are the ideal place to carry out PA promotion interventions. Moreover, there already is sufficient evidence for the increase of PA and fitness in the youth through school-based interventions [89]. However, a process evaluation is essential to control the intervention implementation as well as to examine the range of the program [85]. Otherwise, it is possible that the intervention’s effects are influenced by deficits in execution. For example, Bronikowski et al did not monitor their intervention. Therefore, they argued that it is possible that the participants received homework to reach their goals, which could have influenced the study results as well as the fact that all other daily and weekly activities (eg, PE lessons) continued but were not accurately recorded [70].

These notes could be considered as limitations of both the included studies with the use of wearable activity trackers. If intervention studies use these devices as tools with a focus to facilitate behavior change by motivating and supporting, the limitations may be less of an issue. However, if the studies want to evaluate their outcomes by means of these devices, validity and reliability should be established before such use.

Finally, based on the 2 studies, a clear need for RCTs with longer intervention duration (≥6 months) and sufficient participants is also indicated here. In addition, a follow-up is essential to evaluate the sustainability of using wearable activity trackers. Future research should also be grounded on proved theoretical frameworks to identify the effectiveness of wearable activity trackers for promoting and increasing PA among children and adolescents. In addition, here IM is a helpful planning program framework for development, which integrates, as already mentioned, an evaluation of the intervention program [87].

### Self-Monitoring Using Wearable Activity Trackers

The studies also used their wearable activity tracker to self-monitor PA in combination with various intervention approaches. In both studies, the approach of *goal setting* was used [70,71], which is an effective BCT [82]. This technique was often used in PA interventions, because setting specific difficult goals is suitable to enhance PA levels [90]. If the goals are not difficult enough to reach, as in the study by Bronikowski et al, the effect of changing PA levels could not be significant [70]. However, who set the goals varied in both studies. In 1 study, 1 group had fixed aims of daily steps and the other group did as many steps as they could and wanted. The number of steps was apparent for both groups. Regular support or receipt of tailored advice was not reported [70]. The missing masking of the wearable activity tracker could have represented a motivating factor for the group without fixed aims. The blinding of the monitors could be a benefit for the participants to exploit all their abilities and not just achieve their potential goals [70]. In addition, it has been noted that research has shown that allocated goals are equally effective as self-set goals [91]. In contrast, in the intervention program with elementary school children, Hayes and Van Camp set the goals based on the baseline values monitored and then provided rewards in relation to reaching the goals [71]. Rewarding participants for their achievement of their goals is associated with significantly higher PA effect sizes (*P*<.05) [90].

For future research, it seems to be of great importance to create an incentive for increasing PA in terms of high but achievable goals or also by praising or rewarding participants for achieving their goals or their attempts. Other proved BCTs to increase PA can also be taken into account. Furthermore, the comment by Ridgers et al is appropriate. They demanded to evaluate how children and adolescents engage with wearable activity trackers. Therefore, it can be determined whether the frequency of self-monitoring is mediated by the activity goal [14]. These demands on future research results will show if the approaches will be more effective against the background of this review and provide evidence as to how these technical devices can be successfully integrated into future health promotion interventions to promote PA and to support children and adolescents to reach their recommendations of PA constantly.

### Strengths and Limitations

There are some strengths of this review that should be noted. One of these is focusing on healthy children and adolescents. To date, only a small number of reviews had their focus on this population group without any medical diseases or health restrictions. Another strength is the use of an established evidence synthesis method to evaluate the effects of the tools in the field of mHealth or wearable activity trackers on PA-related outcomes. Moreover, comprehensive conditions have to be fulfilled to achieve high methodological quality. All studies with tools of mHealth could be assessed according to this scheme.

However, there are also some limitations. It is appropriate to include only RCTs in a review. They are considered to be studies with high methodological quality so that their results are particularly meaningful [57]. In this review, several study designs were included that have a methodologically lower quality. This is due to the fact that for the review question about trials with tools of wearable activity trackers, studies with a high-quality design were not available at a preliminary examination. For this reason, these study effects need to be interpreted with caution. Furthermore, only PA-related outcomes were considered and analyzed. However, PA was often not the primary outcome of the included studies; therefore, conclusions on the effect on PA are limited.

### Conclusions

On the basis of the findings of this study, to date, no clear recommendations can be derived. Some studies made restrictions in relation to participants’ PA level so that the populations were not always compared with each other. Moreover, most of the studies based their intervention on several components so that the focus was not only on the mHealth tool or wearable activity tracker. However, as tools in the field of mHealth, mobile games as apps were widely accepted. Future research should focus on developing age-appropriate games to increase PA among children and adolescents. In addition, multicomponent approaches could be more effective in encouraging PA among the youth and should be promoted. A combination of school-based interventions with family or community involvement for social support was applied in some studies and could be an effective strategy.

Overall, the evidence of no effect for intervention studies with tools of mHealth on PA-related outcomes as well as both studies with wearable activity trackers with lower methodological quality shows a clear need for future intervention programs. There is a great lack of studies that seems to exist, especially in the European area. Future studies should be based on IM to develop theory- and evidence-based interventions. By means of this framework, implementation issues are also becoming transparent. Moreover, future studies should aim to strengthen the evidence with a high methodological quality design, an appropriate sample size, a focus on special target groups, follow-up beyond postintervention to assess sustainability, and the use of objective and valid measuring instruments to determine overall activity. In addition, for future transfer of strategies into public health promotion, cost-effectiveness analyses should be carried out.

## Appendix

Multimedia Appendix 1 Search strategy including MeSH (Cochrane Central Register of Controlled Trials, PubMed).

Multimedia Appendix 2 Search strategy excluding MeSH (Scopus, SPORTDiscus, Web of Science).

Multimedia Appendix 3 Criteria for assessment of methodological quality.

Multimedia Appendix 4 Intervention characteristics of included studies aimed to promote physical activity among healthy children and/or adolescents with mHealth tools.

Multimedia Appendix 5 Intervention characteristics of included studies aimed to promote physical activity among children and/or adolescents with wearable activity trackers.

Multimedia Appendix 6 Methodological quality of the selected intervention studies with group comparison.

Multimedia Appendix 7 Methodological quality of the selected intervention studies (number of studies and percentages).

## Abbreviations

BCT: behavior change technique
BMI: body mass index
CENTRAL: Cochrane Central Register of Controlled Trials
CG: control group
CT: controlled trial
IG: intervention group
IM: intervention mapping
MeSH: Medical Subject Headings
mHealth: mobile health
MKMM: MobileKids Monster Manor
MPA: moderate physical activity
MVPA: moderate- to vigorous-intensity physical activity
PA: physical activity
PE: physical education
PRISMA: Preferred Reporting Items for Systematic Reviews and Meta-Analyses
RCT: randomized controlled trial
SMS: short message service
VPA: vigorous physical activity
WHO: World Health Organization
