# Supplementary material for: Effects of Mobile Health Including Wearable Activity Trackers to Increase Physical Activity Outcomes Among Healthy Children and Adolescents: Systematic Review
Source: JMIR Mhealth Uhealth. 2019 Apr 30;7(4):e8298. doi: 10.2196/mhealth.8298 (PMC6658241; doi:10.2196/mhealth.8298)
Supplement: Multimedia Appendix 2 [file mhealth_v7i4e8298_app2.pdf]

Set#	Search string
1 Population	child*[tw] OR adolescent*[tw] OR teen*[tw] OR youth[tw]
2 Treatment method	health promotion[tw] OR health campaign[tw] OR intervention[tw] OR trial[tw] OR program[tw]
3 Treatment objective	*mhealth*<br>mHealth[tw] OR mobile health[tw] OR telehealth[tw] OR eHealth[tw] OR digital health[tw] OR mobile app*[tw] OR mobile phone*[tw] OR smartphone*[tw] OR iPhone*[tw] OR iPad*[tw] OR tablet*[tw] OR android[tw] OR sms[tw] OR text messag*[tw] OR reminder system*[tw]<br>*wearable activity trackers*<br>wearable device[tw] OR wearable act*[tw] OR wearable track*[tw] OR electronic track*[tw] OR electronic activ*[tw] OR health track*[tw] OR FitBit[tw] OR Jawbone[tw] OR Garmin vivofit[tw] OR fitness track*[tw] OR physical fitness track*[tw] OR activity track*[tw] OR activity monitors[tw]
4 Outcome variable	physical activity[tw] OR fitness[tw] OR exercise[tw] OR energy expenditure[tw]
5	(#1 AND #2 AND #3 AND #4)
6	limit #5 to publication dates (01.2012 to 12.2016), English-language, and human

Note: In databases Scopus and Web of Science phrase search with wildcard (")
