# Supplementary material for: Effects of Mobile Health Including Wearable Activity Trackers to Increase Physical Activity Outcomes Among Healthy Children and Adolescents: Systematic Review
Source: JMIR Mhealth Uhealth. 2019 Apr 30;7(4):e8298. doi: 10.2196/mhealth.8298 (PMC6658241; doi:10.2196/mhealth.8298)
Supplement: Multimedia Appendix 3 [file mhealth_v7i4e8298_app3.pdf]

Item	Description
(1) Pre-test analysis	Were the participants' characteristics and central outcome variables analyzed before the beginning of the intervention?
	Were the intervention group (IG) and the control group (CG) comparable at baseline on key characteristics (e.g., age, gender, weight, PA-relevant outcome measures)?
	Were differences between IG and CG in these variables statistically controlled in the further analysis?
(2) Randomization	Were the participants randomized into IG and CG?
	Was the randomization procedure clearly described?
	Did the randomized sample consist of more than 50 participants?
(3) Student drop-out rate	Was the student drop-out rate less than 20% in studies with a follow-up of 6 months or less than 30% in studies with a follow-up of more than 6 months?
(4) Timing of measurements	Did data collection of IG and CG take place in comparable timeframes?
(5) Measure of PA-related outcome	Was a validated measure of PA-related outcome used (reported or referred to)?
(6) Blinding outcome assessment	Were the persons collecting the data not informed about the group membership of the participants?
(7) Follow-up	Was a follow-up measurement realized at a minimum of 3 months after completion of the intervention?
(8) Systematic drop-out	Did the drop-outs not differ from the subjects that completed the intervention?
(9) Sample size	Was the study sample larger than 250 participants or was a power calculation carried out?
