# Supplementary material for: Effects of Mobile Health Including Wearable Activity Trackers to Increase Physical Activity Outcomes Among Healthy Children and Adolescents: Systematic Review
Source: JMIR Mhealth Uhealth. 2019 Apr 30;7(4):e8298. doi: 10.2196/mhealth.8298 (PMC6658241; doi:10.2196/mhealth.8298)
Supplement: Multimedia Appendix 4 [file mhealth_v7i4e8298_app4.pdf]

Table 4. Intervention characteristics of included studies aimed to promote PA among healthy children and/or adolescents with mHealth tools.

Study	Design; country	Recruitment	Participants' characteristics *Number; gender; age; BMI*	mHealth tool	Intervention description	Measures of PA-related outcome	Key findings *Physical activity/fitness*
Dewar et al.[70] (2014)[a]	RCT (cluster randomized controlled trial); Australia	18 public secondary schools in low-income communities invited to participate, 12 randomized by school to IG or CG	*n*=357 girls (IG: *n*=178; CG: *n*=179), 8th grade at time of recruitment (2nd year of secondary school); mean age 13.2 years (SD 0.5); 0.6% underweight, 26.1% overweight, 16.8% obese, BMI *z*-score 0.80 (1.14)	Text message	IG: 12-month multi-component school-based 'Nutrition and Enjoyable Activity for Teen Girls' (NEAT Girls) PA and nutrition intervention consisted several components: enhanced school sport sessions, interactive seminars, nutrition workshops, lunch-time PA sessions, PA and nutrition handbooks, parent newsletters, pedometers for self-monitoring, text messaging for social support<br>Regarding text messaging PA functions: sent weekly (40 weeks) and twice weekly (10 weeks) text messages to encourage to be physically active and also provide strategies to overcome barriers to PA<br>CG: no treatment	Baseline (May/June 2010), 12-months (May/June 2011 - end of intervention) and 24-months follow-up (May/June 2012)<br>Accelerometers:<br>'ActiGraph' accelerometers (MTI models 7164, GT1M and GT3X), should be worn during waking hours for 7 consecutive days<br>Questionnaire:<br>6 social cognitive scales for PA mediators were designed based on constructs of SCT by Bandura: self-efficacy, environmental perceptions, social support, behavioral strategies, outcome expectations and expectancies, intentions to be physically active	A total of *n*=246 met the PA inclusion criteria at baseline and/or 12 months (means 61.9% of the total sample at baseline and 24.6% at 12 months); no significant group-by-time effects on MPA (%), VPA (%) and MVPA (%) in total, during school hours, after school hours and week days ($p > 0.05$); no significant group-by-time effects for all social cognitive variables for PA ($p > 0.05$)
Direito et al.[71] (2015)[a]	RCT (3-arm, parallel, randomized controlled trial); New Zealand	Advertisements in electronic mailing lists, local newspapers, flyers, presentations at schools (recruitment from October 2013 to June 2014)	*n*=51 (IG1 immersive app 'Zombies, Run!': *n*=17; IG2 non-immersive app 'Get Running': *n*=16; CG: *n*=18); 57% female (*n*=29); mean age 15.7 years (SD 1.2, range 14-17 years); BMI 22.85 (4.25), BMI *z*-score 0.60 (1.12)	Smartphone apps	IG1/2: 8-week training program (apps designed to improve fitness and ability to run 5 km), IGs were encouraged to use their app 3 times per week and met each workout, there were no requests to use the app<br>CG: should continue with their usual physical activities, was not offered any information about increasing PA	Baseline and 8 weeks (September 2014)<br>Accelerometer:<br>'ActiGraph' accelerometer (model GT1M) should be worn during waking hours for 7 days after each assessment to evaluate free-living PA, booklet detailing use<br>Questionnaires:<br>For self-reported PA the 'Physical Activity Questionnaire for Adolescents' (PAQ-A) was used, for self-efficacy the 'Physical Activity Self-Efficacy Scale' (PASES) was used<br>Fitness test:<br>1-mile walk/run	No significant intervention effects for self-reported PA (PAQ-A; IG1: adjusted mean difference 0.14, 95% CI -0.26 to 0.54, p=0.78; IG2: adjusted mean difference 0.23, 95% CI -0.18 to 0.64, p=0.42); no intervention effects for PA self-efficacy (PASES; IG1: adjusted mean difference -0.02, 95% CI -0.24 to 0.19, p=0.99; IG2: adjusted mean difference 0.04, 95% CI -0.18 to 0.26, p=0.96); no significant effects on overall activity (average daily time spent in MVPA (min) p=0.98 (IG1), p=0.99 (IG2)); 94% provided valid data for acceleration measurement at baseline, 90% at post-intervention; no significant effects for time to complete fitness test (IG1: adjusted mean difference -28.36, 95% CI -66.54 to 9.82, p=0.20; IG2: adjusted mean difference -24.67, 95% CI -63.51 to 14.18, p=0.32)
Garde et al.[72] (2016)[a]	RCT (randomized controlled cross-over trial); Canada	1 elementary school, grades 4th-5th and 6th-7th	*n*=42; 62% boys (*n*=26); mean age 11.3 years (SD 1.2, range 9-13 years); 2% underweight; 23% overweight, 10% obese, BMI *z*-score 0.28 (1.29)	Smartphone app	4-week game intervention of 'MobileKids Monster Manor' (MKMM) which is wirelessly connected to an accelerometer-based activity monitor	Baseline and 4 weeks<br>Accelerometer:<br>'Tractivity' accelerometer measured continuously PA data (steps and active minutes)	A total of *n*=28 provided valid data for acceleration measurement; game phase showed a significantly greater increase in PA relative to baseline compared to CG (steps/day 95% CI 1,434 to 4,434, p=0.0004; active-min/day 95% CI 20 to 72, p=0.001);

Table 4. (Continued)

Study	Design; country	Recruitment	Participants' characteristics<br>*Number; gender; age; BMI*	mHealth tool	Intervention description	Measures of PA-related outcome	Key findings<br>*Physical activity/fitness*
					Method: *Baseline, game intervention/control, washout*, and *control/game intervention;* each phase being 1 week in duration; all participants had to wear the wireless activity monitor 'Tractivity' for the study duration<br>IG: played MKMM at their convenience, both inside and outside the school environment<br>CG: wore only the activity monitor, no access to the game		boys were significantly more physically active (steps/day p=0.01; active-min/day p=0.02); significant increase in PA at a higher BMI z-score (10% more steps/day, p=0.01; 14% more active-min/day, p=0.002)
Lubans et al.[73] (2016)[a]	RCT (cluster randomized controlled trial); Australia	14 state-funded secondary schools in low-income areas	n=361 boys (IG: n=181; CG: n=180); mean age 12.7 years (SD 0.5, range 12-14 years); 1.9% underweight, 21.3% overweight, 14.4% obese, BMI 20.5 (4.1), BMI z-score: IG 0.64 (0.20); CG 0.51 (0.18)	Smartphone app	IG: 10-week[b] school based intervention 'Active Teen Leaders Avoiding Screen-time' (ATLAS) consisted several components: professional development, fitness equipment for schools, teacher-delivered PA sessions, lunch-time activity sessions, researcher-led seminars, a smartphone app, parental strategies<br>Regarding smartphone app PA functions: PA monitoring through recorded daily step counts from pedometers, recorded and reviewed of fitness challenge results, goal setting for PA, tailored motivational messages over a period of 15 weeks<br>CG: no treatment	Baseline, 8-months and 18-months follow-up<br>Accelerometer:<br>'ActiGraph' accelerometer (model GT3X), worn during waking hours for 7 consecutive days	No intervention effects on PA (percentage of time in MVPA; group-by-time effect p=0.805); 73.4% provided valid data for acceleration measurement at baseline, 53.6% at 8-months, 44% at 18-months
Zach et al.[74] (2016)[a]	RCT (3-arm, parallel, randomized controlled trial)[c]; Israel	1 comprehensive urban school	n=154 high school students (IG1: n=54; IG2: n=46; CG: n=54); ages 16-18 years (mean age not reported); BMI not reported	Smartphone app	12-week intervention program on motivational climate in PE lessons, on female adolescents' self-efficacy for independent PA in leisure time, and on physical fitness measures<br>IG1: exposed to a program to increase motivation for PA in PE lessons and leisure time using group discussion on the school website and weekly self-report about PA submitted to the teachers' website	Baseline and 12 weeks<br>Questionnaire:<br>'Self-efficacy in training' questionnaire to measure confidence to be physically active in the presence of barriers<br>Fitness test:<br>Physical fitness measures (e.g., 12-min run)	'Self-efficacy in training' questionnaire: only significant pre-post test difference in all groups in reported decline of intensity of negative affect towards running (p=0.04); significant differences between groups in all physical fitness measures (p < 0.001); IG1 and IG2 improved to a greater extent

Table 4. (Continued)

Study	Design; country	Recruitment	Participants' characteristics *Number; gender; age; BMI*	mHealth tool	Intervention description	Measures of PA-related outcome	Key findings *Physical activity/fitness*
					IG2: followed the regular PE curriculum, received general guidelines about importance of PA during leisure time, self-report to the class at the end of each lesson about their PE activity using a smartphone app ('WhatsApp')<br>CG: no treatment		

[a] Study with significance level of 5% ($p < 0.05$)
[b] Intervention has subsequently been modified, inter alia, originally designed as an 8-month intervention, on the basis on enhancing sustainability and scalability reducing program duration to 10 weeks
[c] Study design not reported, intervention description contains characteristics of a 3-arm, parallel, randomized controlled trial

Abbreviations: app = application; BMI = body mass index calculated as weight in kilograms divided by height in meters squared,[117] SD in brackets; BMI *z*-score = measures of relative weight adjusted for child age and sex (also called BMI standard deviation scores),[117] SD in brackets; CG = control group; CI = confidence interval; IG = intervention group; MPA = moderate physical activity; MVPA = moderate-to-vigorous physical activity; *n* = number of participants; *p* = test value; PA = physical activity; PE = physical education; RCT = randomized controlled trial; SCT = social cognitive theory; SD = standard deviation; VPA = vigorous physical activity
