# Supplementary material for: Effects of Mobile Health Including Wearable Activity Trackers to Increase Physical Activity Outcomes Among Healthy Children and Adolescents: Systematic Review
Source: JMIR Mhealth Uhealth. 2019 Apr 30;7(4):e8298. doi: 10.2196/mhealth.8298 (PMC6658241; doi:10.2196/mhealth.8298)
Supplement: Multimedia Appendix 5 [file mhealth_v7i4e8298_app5.pdf]

Table 5. Intervention characteristics of included studies aimed to promote PA among children and/or adolescents with wearable activity trackers.

Study	Design; country	Recruitment	Participants' characteristics<br>*Number; gender; age; BMI*	Wearable activity tracker	Intervention description	Measures of PA-related outcome	Key findings<br>*Physical activity/fitness*
Bronikowski et al.[75] (2016)[a]	Before-and-after trial[c]; Poland	Same grade level of standard urban schools in Poznań (data collected in 2015)	*n*=193 (*n*=65 adolescents, mean age 17.2 (SD 0.2), (*n*=35 boys, body mass 71.6 (9.5), BMI 21.5 (2.4); *n*=30 girls, body mass 57.5 (7.8), BMI 20.5 (2.4)); *n*=71 young adolescents, mean age 15.3 (SD 0.2), (*n*=39 boys, body mass 59.2 (9.4), BMI 19.7 (2.4); *n*=32 girls, body mass 58.0 (7.7), BMI 21.5 (2.6)); *n*=57 children, mean age 11.5 (SD 0.4), (*n*=27 boys, body mass 44.4 (10.2), BMI 19.4 (3.3); *n*=30 girls, body mass 42.4 (10.2), BMI 18.7 (2.9)))	Garmin vívofit (model not reported)	8-week intervention<br>IG1 ('Goal set'): daily goal of 10,000 steps (12,000 steps for young adolescents and children)<br>IG2 ('Do your best'): as many steps could and wanted per day<br>IG1/2: could see their number of steps, each participant had an internet account to follow their progress and weekly trends; during intervention, all other daily and weekly activities (e.g. school PE program) were carried out according to the normal routine	Baseline and 8 weeks<br>Wearable activity tracker: 'Garmin vívofit' activity tracker were worn 24 hours a day for 8 consecutive weeks, calculation daily average steps during the intervention (post-test only)<br>Questionnaire:<br>PA subjective screening measure with two questions to evaluate the MVPA index (P1: Over the past 7 days, on how many days were you physically active for a total of at least 60 min per day? P2: Over a typical or usual week, on how many days are you physically active for a total of at least 60 min per day?)	No significant effects between average steps of IG1 and IG2 among adolescents ($p > 0.05$), adolescents of both IGs achieved more than 10,000 steps a day; young adolescents girls of IG1 and IG2 did not met 12,000 steps; only female children of IG2 met 12,000 steps; daily average was in most cases higher in IG2; no interactional effects ($p > 0.05$); significant gender (p=0.003) and group effects (p=0.02) among young adolescents; significant group effects among children (p=0.002); IG1 and IG2 achieved comparable number of steps; significant correlations between pre and post MVPA (r=0.45), IG1 improvement in self-reported MVPA (pre-test M=4.2 (SD 1.46), post-test M=4.3 (SD 1.59))
Hayes et al.[76] (2015)[b]	Before-and-after trial[d]; USA	3rd grade class of an elementary school	*n*=6 girls (part of an intact social group); 8 years old; one underweight, one overweight	Fitbit (model not reported)	Recess intervention (22 sessions in total, 20 min in duration each) with four components: reinforcement, self-monitoring, goal setting, and feedback; 'Fitbit' activity tracker was used to self-monitor PA-levels against set goals; tangible rewards were given if goals met	Baseline and during each of the 22 recess sessions<br>Wearable activity tracker: 'Fitbit' activity tracker used to record the number of steps; before each session, total steps were reset to zero	47% more steps during intervention phases; increasing MVPA from baseline (M=4%) to intervention (M=25%)

[a] Study with significance level of 5% ($p < 0.05$)
[b] No statistical data analyses
[c] According to study description, use of an experimental design with pre- and post-test, on the basis of pre- and post-test placed on the review and classified as a before-and-after trial
[d] Study design not reported, intervention description contains characteristics of a before-and-after trial

Abbreviations: BMI = body mass index calculated as weight in kilograms divided by height in meters squared,[117] SD in brackets; IG = intervention group; *M* = arithmetic mean; MVPA = moderate-to-vigorous physical activity; *n* = number of participants; *p* = test value; PA = physical activity; PE = physical education; *r* = correlation coefficient; SD = standard deviation
