# Supplementary material for: Effects of Mobile Health Including Wearable Activity Trackers to Increase Physical Activity Outcomes Among Healthy Children and Adolescents: Systematic Review
Source: JMIR Mhealth Uhealth. 2019 Apr 30;7(4):e8298. doi: 10.2196/mhealth.8298 (PMC6658241; doi:10.2196/mhealth.8298)
Supplement: Multimedia Appendix 6 [file mhealth_v7i4e8298_app6.pdf]

Study	Item (1)	(2)	(3)	(4)	(5)	(6)	(7)	(8)	(9)	Score	Quality	PA outcome measure
Dewar et al.	1	1	1	1	1	0	0	0	1	6	high	MPA, VPA, MVPA, social cognitive scales for PA
Direito et al.	1	1	0	1	1	0	0	1	1	6	high	PA levels, self-reported PA, PA self-efficacy
Garde et al.[a]	1	0	0	1	1	0	0	0	0	3	moderate	PA (number of steps/day, active minutes/day)
Lubans et al.	1	1	1	1	1	0	1	1	1	8	high	MVPA
Zach et al.	1	0	0	1	1	0	0	1	1	5	moderate	PA self-efficacy, physical fitness

[a] Additional involvement of previous publication to answer the item (9)

Notes: 1 = criterion fulfilled; 0 = criterion not fulfilled or not (sufficient) information given

Abbreviations: MPA = moderate physical activity; MVPA = moderate-to-vigorous physical activity; PA = physical activity; VPA = vigorous physical activity
