# Supplementary material for: Effects of Mobile Health Including Wearable Activity Trackers to Increase Physical Activity Outcomes Among Healthy Children and Adolescents: Systematic Review
Source: JMIR Mhealth Uhealth. 2019 Apr 30;7(4):e8298. doi: 10.2196/mhealth.8298 (PMC6658241; doi:10.2196/mhealth.8298)
Supplement: Multimedia Appendix 7 [file mhealth_v7i4e8298_app7.pdf]

Item	Criterion fulfilled (1)		Criterion not fulfilled (0)		Not sufficient information given (0)	
(1) Pre-test analyses	5	(100%)	-	-	-	-
(2) Randomisation	3	(60%)	1	(20%)	1	(20%)
(3) Student drop-out rate	2	(40%)	3	(60%)	-	-
(4) Timing of measurement	5	(100%)	-	-	-	-
(5) Measure of PA-related outcome	5	(100%)	-	-	-	-
(6) Blinding outcome assessment	-	-	3	(60%)	2	(40%)
(7) Follow-up	1	(20%)	3	(60%)	1	(20%)
(8) Systematic drop-out	3	(60%)	2	(40%)	-	-
(9) Sample size	4	(80%)	-	-	1	(20%)
